# Understanding and managing somatoform disorders: Making sense of non-sense

**DOI:** 10.4103/0019-5545.69239

**Published:** 2010-01

**Authors:** Roy Abraham Kallivayalil, Varghese P. Punnoose

**Affiliations:** Department of Psychiatry, Co-operative Medical College, Cochin - 683 503, India; 1 Department of Psychiatry, Govt Medical College, Kottayam - 686 008, India

**Keywords:** Somatoform disorders, Medically unexplained somatic symptoms, Management, Positive explanation

## Abstract

Somatization is a clinical and public health problem as it can lead to social dysfunction, occupational difficulties and increased healthcare use. Hence understanding somatoform disorders is of paramount importance, especially so in developing countries like India. This paper discusses the history and evolution of the concept of somatization and somatoform disorders, etiological considerations, classification, assessment, diagnosis and clinical management. Research from India, controversies and criticisms and future perspectives are mentioned. A new model to understand functional somatic symptoms, in Indian setting is also proposed.

“*I am more sick than my doctors think*”

*Alfred Nobel*

## INTRODUCTION

Somatization is a poorly understood “blind spot” of Medicine.[1] Somatoform disorders remain neglected despite functional impairment and economic burden. Conceptual and clinical questions exist about the validity and utility of the concepts. New paradigms might lead to more effective management.[2]

In somatoform disorders, physical symptoms suggest a physical disorder, but there are no demonstrable organic findings and there is strong evidence for link to psychological factors or conflicts. The term is from Greek, “soma” for body. In the middle ages, these disorders were believed to be spiritual disorder of evil and demonic possession. In the 17^th^ century, Sydenham said, “hysteria could simulate any medical disease”. In the 19^th^ century, it was Briquet who made the first systematic description of hysteria with 430 cases. Briquet, Reynold, Charcoat all believed that hysteria is a CNS disease. ‘Studies on Hysteria’ (1893-95) by Breur and Freud gave new insights. Freud explained the syndrome of hysteria as “conversion of emotional distress into physical symptoms”. Later, hysteria became less popular as a diagnosis. The term ‘somatization’ was introduced by Stekl to denote “the expression of emotional distress as bodily symptoms”. Hysteria has pejorative associations. It might represent misdiagnosis of organic disorders.[3] It was the St Louis group Perley and Guze[4] who described the Briquet’s syndrome as “chronic multiple somatic symptoms, with no identifiable organic cause”. They had regarded this as a form of hysteria.

A similar syndrome was described as somatization disorder in DSM III. The term somatoform disorders was introduced in DSM III for “a group of disorders characterized by physical symptoms, not explained by organic factors”. This new category included traditional psychiatric disorders like hysteria and hypochondriasis, together with newly proposed categories like somatization disorder. Somatoform disorders and dissociative disorders were introduced in DSM III “to rationalize what has been previously regarded as neurosis”. These groupings were tentative, lacking substantial evidence base and unsatisfactory.

According to DSM IV, in somatoform disorders the common feature is “presence of physical symptoms which suggest a general medical condition and are not fully explained by general medical condition, substance use or another mental disorder”. This disorder also produces clinically significant distress or impairment in social, occupational or other important areas of functioning. Symptoms are not intentional, contrasting it with factitious disorder or malingering. Conversion disorder is placed in the somatoform section to consider neurological or medical conditions in differential diagnosis. However, the evidence base for diagnosis and treatment remains sub-optimal.

### Conceptualizing somatoform disorders

Somatization can be conceptualized as a process which appears fundamentally as a way of responding to stress. Another concept is somatosensory amplification, where somatic symptoms are experienced as intense, noxious or disturbing.[5] It has three elements i) hypervigilance (to bodily sensations) ii) selecting out some sensations (which are weak) and iii) intensification by cognition and affect, making them more alarming.

Somatization is a clinical and pubic health problem as it can lead to social dysfunction, occupational difficulties and increased health care use. Somatization can also be viewed as masked psychiatric disorder (eg: Depression or Anxiety) or amplified personal perceptual style (due to personality trait or abnormal neuro- psychololgical information processing) or as seeking care for emotional distress or as a response to health care incentives (iatrogenic somatization).

Etiological considerations include patho-physiological mechanisms, genetic and developmental factors, cognitive theories, personality characteristics, psychodynamic factors, sexual and physical abuse, socio-cultural factors, gender and iatrogenesis. Patho-physiological mechanisms can be physiological, psychological and inter-personal. Postulated physiological mechanisms are autonomic arousal, muscle tension, hyperventilation, vascular changes, and cerebral information processing and sleep disturbance. Among psychological mechanisms, perceptual factors, beliefs, mood and personality factors are important. Significant interpersonal mechanisms include re-inforcing actions of relatives and friends, health care system and disability benefits.

Classification: In DSM IV, Somatoform disorders include somatization disorder, conversion disorder, pain disorder, hypochondriasis, body dysmorphic disorder, undifferentiated and somatoform disorder - NOS. In ICD(10), body dysmorphic disorder is included under hypochondriasis and also there are categories for somatoform autonomic dysfunction and other neurotic disorders like neurasthenia. The present classification of somatoform disorders has been criticized by many. Important criticisms are i) mixture of principles for diagnostic criteria (eg: etiology, symptom count and response to treatment) ii) non-specific categories iii) categories are broad and vague iv) possibility to use them for nearly all persistent medically unexplained symptoms and v) leads to large discrepancies in prevalence.

Somatization disorder is chararacterized by multiple somatic symptoms of long duration beginning before the age of 30 years. This was called Briquet’s syndrome (1962) earlier. A similar syndrome was named as somatization disorder in DSM III (1980). The diagnostic criteria were highly restrictive in DSM IIIR.[6] DSM IV[7] made the criteria less restrictive.

### Understanding functional somatic symptoms in Indian setting - A new model

In modern medicine, clinical disciplines traditionally consider only those symptoms which are associated with a physical sign or a laboratory finding as significant. Even when these are absent, a pathophysiology, which is known or at least presumed, may also lend some respectability for the symptoms. For example, complaints like weakness of limbs when associated with changes in deep tendon reflexes or chest pain when ischemic changes in ECG are demonstrated or headache descriptions suggestive of vascular origin or fatigue when investigated shows high TSH values are readily accepted as genuine symptoms. In training undergraduates and post graduates in clinical sciences, only those cases with demonstrable findings and laboratory evidences are thought to be worthy of any serious diagnostic considerations. Hence it is not surprising that they are never taken up for a bedside discussion or a case conference. But in actual clinical settings, either in general practices or in specialty settings, the situation is quite different. A significantly high proportion of patients present with complaints which are not justified by the presence of a corresponding physical sign or a laboratory finding.

As far as the management of these patients is concerned, initially investigations are suggested with enthusiasm and curiosity from the part of the physician to unearth an elusive mysterious diagnosis. Many young doctors are carried away by the medical fairy tales of great clinicians of yesteryears (either intuitively or by ordering for an extraordinary investigation) making rare diagnoses, which has eluded the less diligent eyes of the lesser mortals! But as the patient continues to present with new set of symptoms in every visit or persist with the same complaints and the investigations continue not to reveal anything significant, the initial interest and enthusiasm gives way to frustration and helplessness. This can lead on to unpleasantness and loss of trust in the doctor-patient relationship. The patient who initially had complied to every suggestion for a new investigation or procedure with a hope of getting a diagnosis may start accusing the doctor of forcing unnecessary investigations (with ulterior motives)!

The physician on the other hand may try to believe and explain the symptoms as resulting from vague constructs like allergy, wear and tear due to ageing, psychosomatic, perimenopausal symptoms etc. He may have ambivalent feelings about the possibility of missing a real problem. A physician is also likely to experience guilt over his inability to help the patient, over his incompetency as a clinician, and over the expenses which the patient had to incur. As a reaction formation to these feelings, she may start seeing the patient as a malingerer who is eating up her valuable time and using up the limited medical resources unnecessarily. On the patient’s side also the emotions can run high. He feels betrayed and not cared by his doctor. On the relentless pursuit of finding a meaning to his symptoms he may start believing in equally imprecise constructs like low blood pressure, high ESR, eosinophilia, which may be inadvertently and covertly agreed by the physician who is equally, if not more, at a loss to explain the symptoms.

Unfortunately, in India, undergraduate and post graduate medical and psychiatric training is grossly inadequate to understand and effectively deal with these cases which are considered to be functional! This article is written in the background of clinical experience of the authors in a consultation -liaison setting of a general hospital psychiatry unit. The reasons for medically unexplained physical symptoms remaining one of the areas least explored despite their common occurrence may include the following.

1. Traditionally these conditions are not considered part of core psychiatry. The nosology and classification of these conditions are confusing and controversial.
2. Psychiatrists who are trained in mental hospitals are least exposed and not having adequate expertise in this area.
3. Reluctance from the part of patients to seek psychiatric help.
4. Difficulties encountered by general practitioners and specialists in making a referral for psychiatric help.

Authors from the West have proposed many models in understanding functional somatic symptoms. For example, Linda Gask[8] described a practical model for the detection, acknowledgement and management of these conditions which can be easily learned and used in primary care. The three stage model emphasizes the importance of shared care between the psychiatrist and the primary health care team. These models are either directed to psychiatrists or primary care physicians.[9,10,11,12] Incorporating the research from the developing world, WHO also has come out with training packages addressing the need of such communities.[13] Each of these models has their own strengths and problems. Every centre need to develop and evolve models suiting their needs and limitations as there cannot be a single ideal universal algorithm which may suit a condition as complex and diverse as functional somatic symptoms.

We propose a model for understanding functional somatic symptoms, which is expected to be friendly to the non- psychiatrist users. The non- psychiatrist medical professional may consider the following possibilities when he is encountering medically unexplained somatic symptoms.

1. Symptoms which are in excessive (disproportionate to) the “real disease”
2. Anxiety disorders and Depressive disorders presenting with physical symptoms
3. No known physical or common mental disorders to account for the somatic symptoms
4. Acute and dramatic presentation of physical symptoms without a medical cause
5. Concern and conviction of a disease when none exists
6. Deliberate feigning of diseases

#### Symptoms which are in excessive (disproportionate) to the “real disease”

This constitutes one of the most frequently encountered situations in clinical practice. This pattern of excessive complaining and dissatisfaction may baffle and annoy the physician who expects the patient to show a corresponding improvement directly proportional to the physical signs and lab reports. This may be due to the following factors.

- Normal regressive behavior associated with any medical illness
- Propensity of certain temperamental - personality types to perceive symptoms in high intensity
- Autonomic responses associated with anxiety leading to physical symptoms
- Reward-punishment contingencies perpetuating illness behavior

#### Anxiety/depressive disorders presenting with physical symptoms

The conventional view is that it is the psychological symptoms and not physical symptoms which constitute the legitimate presentation of these emotional disorders. The neurobiological basis of Anxiety disorders and Depressive disorders points to the involvement of dysfunctional serotonergic, nor-adrenergic or dopaminergic neuronal circuits. If the physician realizes that the same dysfunctional circuits can produce “real” physical symptoms and they are thus legitimate manifestation of disorders which are primarily emotional, that will give an explanatory model which will not produce cognitive dissonance to a medically trained mind. Thus he will consider anxiety disorders and depressive disorders higher up in the priority list of differential diagnosis putting them much ahead of rare conditions l

#### No physical or mental disorders to account for the somatic symptoms

When the somatic symptoms cannot be explained based on the above mentioned situations, they become much more difficult to be understood. Even the psychologically minded physicians find it difficult to empathize with this group. Medical professionals not trained in psychiatry may find it very difficult to understand the subtle difference between these disorders and conditions which are of factitious nature. One has to admit that these patients constitute the group which is difficult to tag a diagnosis and manage in the usual way, by virtue of the very nature and chronicity characteristic of these disorders. Naming them as somatoform disorders or sub typing them into somatization disorder or pain disorder may help to differentiate them from malingering or factitious disorders but may not help much in understanding or managing them. But if the physician can understand that these disorders are a result of abnormal processing and perception of signals in the central nervous system, it may help not only them, but also the patients or their worried relatives to make sense out of this baffling presentation.

#### Acute and dramatic presentation of physical symptoms without a medical cause

A psychiatrist may label them as a conversion disorder or a dissociative disorder when such disorders are presumed to have a causal relationship to a psychological conflict which may be unconscious. When he makes a referral to a psychiatrist, a medical professional is usually not bothered over these subtleties and is worried whether he is missing an organic cause, is concerned about symptom removal, and is often curious about the psychological stressor identified. Very often, experienced physicians have evolved their own method of dealing with “hysterical” cases from their experience and not from any formal psychiatric training. One has to admit that these methods are effective at least for symptom removal. The practical difficulties in referring these patients for a psychiatric consultation often cited by physicians are also very valid in the background of our cultural context.

#### Concern and conviction of a disease when none exists

For the psychiatrist, this group whose main concern is not the symptoms, but the beliefs about health, disease and diagnosis may be hypochondriasis, a sub type of somatoform disorder. Understanding the relationship between health anxiety and beliefs about diseases and ill health may provide better insight for the physician in empathizing with these patients who are very likely to elicit negative emotional responses from the therapist and other care takers.

#### Deliberate feigning of diseases

The subtle difference between factitious disorders and malingering does not bother the non-psychiatrist. The fact that these disorders are relatively rare compared to the more common place conditions described above should be imparted to them rather than heading for the hair splitting arguments over factitious versus malingering.

These six situations need not be considered essentially in the exact order given as above. The priority in this article has been assigned depending on the frequency usually encountered in clinical practice in a general hospital setting.

We have abided by the clinical dictum that ‘*uncommon presentations of common conditions are much more common than common presentation of rare conditions*’ in assigning this priority. The clinician should use his practical wisdom in determining priorities in individual cases.

#### Assessment and diagnosis of somatoform disorders

Building an alliance with the patient, collaborating with referral source, reviewing the medical records, gathering collateral information from others, performing psychiatric examination and MSE and physical examination are integral to a proper diagnosis.

#### Clinical management

Adopting ‘caring rather than curing as the goal’ is useful. Management strategies include i) Re- attribution approach ii) Pychodynamic approach and iii) Directive approach. In re-attribution approach the patient is helped to link his physical symptoms with psychological or stressful factors in his life. This is useful in those patients with insight, in short duration illness and for use in PHCs. In the psychotherapeutic approach, the thrust is in forming a close and trusting relationship with the patient. This modality may be useful in persistent somatization. In directive approach, the patient is treated as though he has a physical problem. Interventions are framed in the medical model. This approach is useful in hostile patients and those who deny the relevance of social or psychological factors.

Principles of management are fundamentally same for management of all somatoform disorders. They are:

i) Providing a positive explanation for the symptoms, without dismissing them. Symptoms are to be seen as real and the physician has to appear as one who is keen to explore all possibilities for symptom removal ii) Ensuring regular follow-u p (and not ‘symptom-driven’ visits) iii) Treating mood or anxiety disorders iv) Minimizing polypharmacy v) Providing specific therapy (eg: physiotherapy to reduce musculos- keletal pain) vi) Changing social dynamics that re-inforce the symptoms vii) Emphasizing doctor- patient relationship viii) Recognizing counter-transference ix) Suggestions and reassurance. Explanations are given to empower the patient, emphasizing good prognosis and ensuring active involvement of the patient and x) Specific treatment models like pharmacotherapy, behavior treatments including cognitive therapy and CBT, dynamic psychotherapy, group therapy, marital therapy, family therapy, physical and relaxation therapies. Amalgamating the reviews by O’Dowd (1988), Bass and Murphy,[11] Goldberg *et al*.,[13] and Bass and Benjamin[10] many principles can be suggested in the management of chronic somatization in primary care.

Research from India: Chandrasekhara R *et al*. (1964) found, hysterical neurosis as one of the commonest mental disorders in India. Of the 38 women followed up for five years, 63% remained asymptomatic. Raguram R *et al*.[14] found stigma is positively correlated to depressive symptoms and negatively to somatoform symptoms. Chaturvedi *et al*.[15] designed a screening test of abnormal illness behavior in patients with somatic symptoms -SIBQ- which was useful for busy centers. Nambi SK *et al*.[16] found primary care patients believed in the physical nature of the complaint and in its serious nature. Hence understanding patient’s perspective becomes important. Patel V (2005) found stress as common attribution for vaginal discharge. In such patients there were high scores for somatoform disorders and CMD. Malhotra S *et al*.[17,18] found, in children and adolescents, somatoform disorders and dissociative disorders were closely linked. Age at presentation and intelligence were significantly higher in somatoform disorders. Trivedi JK *et al*.[18] found somatization patients may have substantial cognitive deficits, especially in executive functions, attention, concentration and memory. This might lead to poor psychosocial functioning. Paralikar VP *et al*.[19] studied biomedical markers and psychiatric morbidity in neurasthenia spectrum disorders and found anxiety and somatoform disorders were more frequent than depressive disorders.

Controversies and Criticisms: Some of the important ones about Somatoform Disorders are i) Dualistic explanation sees mind and body exclusively ii) There is dichotomy into psychogenic and somatogenic categories iii) ‘Psychogenic in origin’ is implied in the terminology iv) Many cultures including Indian, do not share the western concept of mind body dualism v) There is lack of clear operational definitions and vi) This seems to be an artificial grouping of conditions and the basis may be ‘psychiatric disorders presenting in Medicine’. There is no doubt, some of these criticisms are valid and it should focus our attention on improving the classification in future.

Future Perspectives: Hypochondriasis and somatization are so enduring and is it more appropriate to classify them as personality disorders? Also, physical and psychological factors contribute to the illness. Hence the dualistic view is likely to be rejected in future. Also there is increasing acceptance that pain cannot be meaningfully classified as either somatogenic or psychogenic.[20,21] And co-morbidity of somatoform disorders with depression and anxiety need to be important considerations for the future.

## CONCLUSION

Managing *FuSS (Functional Somatic Symptoms)* patients is a challenging task for any physician. First and foremost, it is crucial to accept the real nature of the symptoms, with the exception of factitious disorders. Giving an explanatory model for the patient for his symptoms is very important. A patient with medically unexplained somatic symptoms is often at a loss to understand the why and how of his symptoms. Quite often, he is given vague and contradictory explanations which may not be suiting his belief systems and thinking. Prescriptions of psychotropic medicines given without a convincing explanation are very likely to be perceived by the patient as dishonest.

A model which focuses on dysfunctional neurotransmission and brain circuits which are influenced by external stressors and internal conflicts leading on to symptoms may be appropriate in the first three situations. Abnormal signal transmission and processing in the nervous system may also be brought in as legitimate explanations for these conditions. When pharmacological agents are prescribed, they should be explained as agents to correct these irregularities and not as tranquillizers. One cannot expect to make every doctor skilled in individual psychotherapies, but basic principles of behavioral management, counseling and communication skills can be imparted to every medical professional.

Making psychiatry a compulsory subject with at least six weeks of clinical training and examination is likely to equip any doctor with these skills. Teaching of psychiatry at postgraduate level of every clinical subject should also be seriously considered. In post-graduate psychiatric training and examination, the importance given to consultation- liaison psychiatry should be enhanced to meet the challenges and needs in this area. The consultation- liaison work between psychiatrists and specialists in other clinical subjects should be strengthened. Only with these policy changes, the medical profession will be able to meet the unseen but vast need in healthcare.

“*Writing prescriptions is easy, understanding people hard!*”

*Franz Kafka*
